# Education Research: Utilization of Simulation in Residency Programs: A Survey of Adult Neurology Residency Program Directors

**DOI:** 10.1212/NE9.0000000000200156

**Published:** 2024-11-14

**Authors:** Shivani Ghoshal, Catherine S.W. Albin, Nisha A. Malhotra, Ifeyinwa Asonye, John Budrow, Rebecca Stainman, Arielle Kurzweil, Michelle Bell, Jenna Ford, Inna Kleyman, Nina Massad, Yara Mikhaeil-Demo, Briana Wasserstrom, Zahari Tchopev, Nicholas A. Morris

**Affiliations:** From the Department of Neurology (S.G., I.A., J.B., M.B., I.K.), Columbia University Vagelos College of Physicians and Surgeons, New York, NY; Department of Neurology (C.S.W.A.), Emory University School of Medicine, Atlanta, GA; Department of Neurology (N.A. Malhotra, A.K.), NYU Grossman School of Medicine, New York; Department of Neurology (R.S.), Johns Hopkins School of Medicine, Baltimore, MD; Department of Neurology (J.F., Y.M.-D.), Northwestern University Feinberg School of Medicine, Chicago, IL; Department of Neurology (N.M.), University of Miami Leonard M. Miller School of Medicine, FL; Department of Neurology (B.W.), University of Florida College of Medicine, Gainesville; Department of Neurology (Z.T.), F. Edward Hebert School of Medicine, Uniformed Services University, Bethesda, MD; and Department of Neurology (N.A. Morris), University of Maryland School of Medicine, Baltimore.

## Abstract

**Background and Objectives:**

Previous research has demonstrated that simulation-based medical education (SBME) can improve neurology trainees' confidence, knowledge, and competence. However, a general needs assessment and review of current SBME used within neurology are needed to guide SBME curriculum development. The objective of this study was to describe the current use of SBME in resident education and to assess perceived barriers to expanding SBME interventions.

**Methods:**

We surveyed adult neurology residency program directors (PDs) listed in the Accreditation Council for Graduate Medical Education directory using a Qualtrics-based survey platform. Survey questions addressed current utilization of SBME and barriers to SBME growth.

**Results:**

Seventy-five PDs of 171 contactable PDs responded to our survey (response rate 44%). Of the respondents, 84% (64/75) report using SBME in their adult neurology residencies. Of those using SBME, 87% (55/64) programs create their own cases. Most programs use simulation to teach neurocritical care topics (63%) and vascular neurology (78%); few use simulation to teach outpatient topics and teleneurology. Among programs that use SBME, there was variability in the frequency of the SBME interventions and in the target trainee cohort. Among responding programs, most expressed interest in expanding SBME in their curriculum (69%, 52/64), but frequently cited lack of faculty protected time (55%), funding (35%), and resident availability (32%) as barriers to doing so.

**Discussion:**

Most responding programs use SBME. However, the frequency and target learner for SBME interventions varied between programs. Many programs wish to expand SBME at their institutions but are constrained by limited protected time and institutional financial support. We discuss potential solutions to the perceived barriers to SBME, including intra-institutional collaboration to advance SBME use and case diversity for learners and help innovate neurology medical education.

## Introduction

Simulation-based medical education (SBME) has been lauded for its potential to transform health care and patient safety when integrated into graduate medical education.^1^ Through SBME, participants engage in experiential learning followed by a structured debriefing. This leverages the Kolb's theory of learning in which the learner can leverage a concrete experience with reflective observation allowing for abstract conceptualization and active experimentation.^2^ Simulation has been used to teach clinical skills and nontechnical skills including emotional intelligence, crisis resource management, and leadership.^3-5^

A 2010 Association of American Medical Colleges (AAMC) online survey of teaching hospitals reported that less than 10% of programs reported using SBME for neurology education. Since that survey, there has been a dramatic rise in publications about simulation in neurology—a recent scoping review of innovations in clinical neurology education found that more studies of SBME were published over the past decade than any other form of education innovation.^5^ The recent growth of neurology SBME scholarship suggests a rise in SBME implementation within modern neurology training programs. However, to date this has not been objectively demonstrated. To address this gap, we sought to quantify the use of SBME in adult neurology programs and describe how SBME is used in neurology resident education. Our primary goal was to summarize the current use (purpose, content, and frequency) of SBME in Accreditation Council for Graduate Medical Education (ACGME)–accredited adult neurology residency training programs.

Simulation is a resource intensive pedagogy often requiring a high number of participants and faculty (some with some specialized knowledge of case planning and debriefing) and potentially unique training environments, equipment, and time. All these factors have the potential to make simulation curricula more difficult to implement than traditional lecture-based didactics. However, it was not clear which of these factors, if any, are barriers to simulation in neurology programs. Thus, In addition to assessing the use of SBME in adult neurology programs, we also sought to assess program directors (PDs)' interest in expanding SBME in their residency program and the perceived barriers to doing so. We specifically assessed the PDs' interest in collaborations that could offload effort in SBME curricula creation.

## Methods

### Survey Development

To assess the current state of SBME in adult neurology training programs, we created and distributed a survey to all neurology residency PDs listed through the ACGME neurology residency directory (eAppendix).

To ensure clarity of the questions asked, survey questions and listed barriers to SBME implementation were developed through an iterative process, peer review, and eventual consensus among a nation-wide writing group of 15 leading SBME-based educators and trainees within neurology, with 3 meetings over 3 months. Writing group members were identified based on SBME training, research, and experience. The survey was created using the online Qualtrics platform. Questions were organized into multiple sections exploring descriptions of the training, assessment of current SBME utilizations, barriers to SBME utilization, and interest in growth of SBME at each training program. Free-text options for simulation purpose, case topics, and barriers to SBME utilization were included for any areas not identified by the survey writing group. Respondents were able to select multiple answers per section.

**Figure 1:**
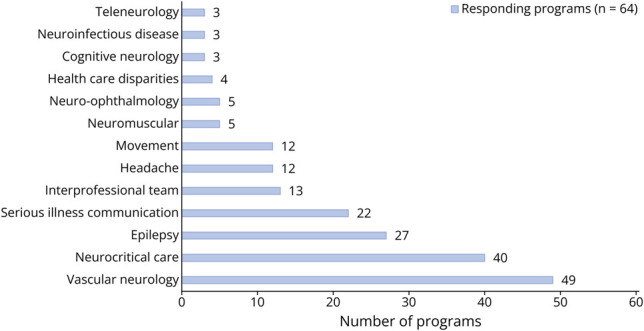
Simulation Case Topics Among Simulation-Based Medical Education Neurology Programs

Program descriptors included program size, frequency of simulation, target learners in simulation, and purpose of simulation. The purpose of simulation was subdivided as education, assessment, interprofessional development, quality improvement, or trainee clinical performance. Trainee performance as an assessment measure was listed as a separate purpose from research of trainee behavior in survey prompts. Simulation participation among faculty, nursing, advanced practice providers, and pharmacy were collected as further descriptors for interprofessional simulation. Respondents could select simulation topics established at their training program, detailed in Figure 1. Barriers to SBME utilization were divided as constraints on faculty time, faculty training, curriculum funding, case availability, collaboration availability, simulation center and equipment availability, resident availability, mentorship, and previous experience in simulation. The survey closed with an assessment of sources for simulation cases, interest in a centralized source for neurology simulation cases, and interest in further growth of the program's simulation curriculum.

### Standard Protocol Approvals, Registrations, and Patient Consents

The study was approved by the Columbia University Irving Medical Center institutional review board, including waiver of written informed consent.

### Settings and Participants

All ACGME-accredited US adult neurology residency PDs were identified as survey participants. Email invitations to participate were sent to each PD's ACGME-listed email in November 2022. Responses were collected up until March 2023. Three follow-up emails were sent to nonresponders between November and March.

### Data Analysis

Program characteristics were compared as descriptive data. Median program sizes were compared between programs, as residency sizes varied widely among survey respondents and the distribution was not normal. A portion of survey responses were incomplete, particularly among programs not using SBME. All completed responses were included in analysis, leading to differing denominators of total responses based on section, as reflected in Tables 1–3. Survey sections that were not completed were not included in the corresponding sections' analyses.

**Table 1:** Neurology Residency Program Characteristics

No. of invited programs	171
Total survey respondents, n (%)	75 (44)
Programs with SBME, n (%)	64/75 (85)
Median size of program with SBME, median (IQR)	24 (6–44)
Interest in incorporating more SBME into program curricula, n (%)	52/64 (81)
Programs without SBME, n (%)	11/75 (15)
Median size of program without SBME, median (IQR)	23 (12–24)
Purpose of simulation in programs with SBME (total 64 respondents)	
Trainee education, n (%)	57/64 (89)
Trainee performance assessment, n (%)	32/64 (50)
Interprofessional development, n (%)	21/64 (33)
Trainee behavior, n (%)	13/64 (20)
Quality improvement, n (%)	7/64 (11)
SBME case development in programs with SBME	
Program writes own cases, n (%)	55/64 (86)
Program collaborates with other institutions, n (%)	10/64 (16)

Abbreviations: IQR = interquartile range; SBME = simulation-based medical education.

**Table 2:** Characteristics of Simulation Curriculum in Neurology Programs With SBME

Simulation participants (total 64 respondents)	n (%)
Neurology resident, postgraduate year 2	50/64 (78)
Neurology resident, postgraduate year 3	34/64 (53)
Neurology resident, postgraduate year 4	30/64 (47)
Pediatric neurology resident	16/64 (25)
Fellows	16/64 (25)
Faculty	15/64 (23)
Nursing	10/64 (16)
Advanced practice providers	7/64 (11)
Pharmacy	1/64 (2)
Frequency of simulation (total 58 respondents)	
Monthly	1/58 (2)
Few times a year	40/58 (69)
Once a year	17/58 (19)

**Table 3:** Interest in Neurology SBME Case Repository Among Programs Utilizing SBME

Total number of programs utilizing SBME	64
Interest in participation in neurology SBME case repository, n (%)	63/64 (98)
Most common requested repository topics (total 24 respondents), n (%)	
Neurologic emergencies (ICP crisis, stroke code, status epilepticus, cord compression)	9/24 (37)
Serious illness conversation	9/24 (37)
Brain death	8/24 (33)

Abbreviations: ICP = intracranial pressure; SBME = simulation-based medical education.

### Data Availability

Anonymized data not published within this article will be made available by request from any qualified investigator.

## Results

### Overall Results

A total of 175 adult neurology residency programs were identified, with 4 programs excluded because of lack of listed contact email, and the survey was emailed to 171 ACGME-accredited US adult neurology residency PDs. The response rate for the survey was 44% (75/171). Program characteristics of responding PDs are described in Table 1.

Of the programs that responded, 85% (64/75) reported use of simulation training in their residency programs. Median size of programs (24 residents) was similar between programs that did and did not use SBME. Most (55/64, 86%) programs using simulation reported creating their own individual simulations. The remainder of programs reported collaboration with other programs or online sources for their simulations. Of responding programs currently using SBME, 81% (52/64) of programs reported interest in incorporating more simulation training into their institutional curricula.

Most (57/64) responders felt the purpose of simulation at their respective institutions was primarily to provide trainee education, followed by assessment of trainee performance and support of interprofessional development. Simulation was less commonly used as assessment of trainee behavior, and few programs used simulation to inform quality improvement projects.

In programs using simulation, learners in programs using simulation were primarily first-year neurology residents (50/64); further characteristics are described in Table 2. Simulations were performed once a year in a quarter of responding programs and “a few times throughout the year” for the remainder. Neurologic emergencies were the most common topic for SBME, with 78% of programs with simulation cases for vascular neurology and 63% with cases in neurocritical care. Health care disparities/implicit bias and teleneurology were the least common simulation topics.

Of the responding programs currently using SBME, 98% (63/64) expressed interest in a neurology simulation case library, with specific interest in topics of brain death, coma, procedural skills, communication strategies (breaking bad news, disclosing medical errors, dealing with difficult patients), status epilepticus, stroke, and management of intracranial pressure crisis. Responses to the survey's free-text options largely focused on requested case topics for the simulation case library, as summarized in Table 3.

### Barriers to Use of Simulation

**Figure 2:**
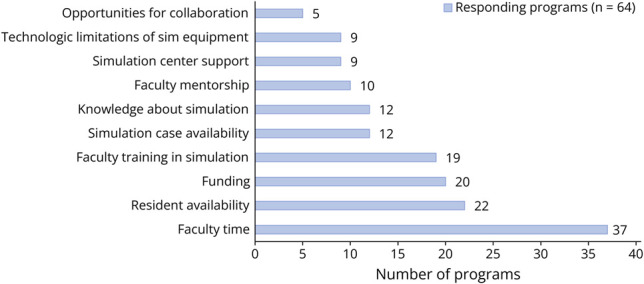
Barriers to Simulation Growth in Neurology Programs Currently Using Simulation-Based Medical Education

Among programs using SBME, reported barriers to greater utilization of simulation in educational initiatives are primarily related to limitations in faculty protected time for 41 of 75 (55%) responding programs, lack of funding and departmental support in 26 of 75 (35%) responding programs, and limitations in resident availability to participate in 24 of 75 (32%) programs. Other limitations noted by responding programs include lack of faculty training in simulation, lack of prewritten case availability, and limited mentorship and avenues for publication. Only 10 of 75 (13%) programs reported lack of support from simulation center staffing as a barrier (Figure 2).

## Discussion

Our survey of 75 adult neurology PDs found that most responding residency programs incorporate simulation, albeit only a few times per year. Most programs use simulation primarily as a clinical education tool targeting junior residents. SBME was most often used to teach neurologic emergencies and was rarely used to teach outpatient topics. Few programs use simulation to assess resident competency, provide interprofessional education, or engage in quality improvement. Despite commonly shared topics within SBME, most programs continue to create their own simulation cases.

Our results build on a 2010 AAMC online survey which found that most teaching hospitals (55/64) incorporated SBME. However, at that time, only 10% of teaching hospitals used SBME to teach neurology.^6^ Our results suggest that SBME has increased dramatically over the past decade within neurology residency programs. The same AAMC survey found that SBME within teaching hospitals particularly focused on the valuation, certification, and remediation of psychomotor skills, especially in regard to credentialing of procedural privileges.^7^ Neurology residency programs may have been slow to implement SBME as neurology training has not traditionally focused on procedural learning. The expansion of modern SBME focuses beyond technical skills and thus has been more easily integrated into neurology residency training.^8^

Our results show that SBME is currently most often used to teach the management of vascular neurology and neurocritical care topics to junior residents. Although this is an important application of SBME, there are many other potential ways that simulation could be incorporated into resident training. For example, SBME can be leveraged to give feedback on delivering serious medical news and incorporating shared decision making—skills that are both important in the inpatient and outpatient settings.^4,7-13^ Furthermore, our survey demonstrated that faculty, nursing, pharmacy, and advanced practice providers are rarely incorporated into neurology resident SBME experience. This suggests that currently simulation is not being fully leveraged to teach interprofessional teamwork and communication skills, core competencies for ACGME neurology residency training.^10,11,13^ Simulations involving both faculty and residents may offer insight into professionalism and communication within medical team hierarchies. Finally, our survey results demonstrated that quality improvement is least often the focus of SBME within responding neurology programs. Other specialties have used SBME to teach root cause analysis.^14^ Expansion of SBME outcomes beyond trainee learning to time to care delivery, patient outcomes, or reduction of medical errors may advance SBME as a quality improvement tool within neurology resident training.^12,13^

Programs may already recognize the myriad ways in which SBME could enhance training. Of those that currently use simulation, most agreed they wanted to incorporate more SBME. However, faculty time, resident availability, and funding were listed as the greatest barriers to SBME growth. Limited mentorship, faculty training, and simulation case availability were also cited as barriers, but these were reported less frequently. These findings echo those from an international survey of 42 health care education institutions with simulation centers that found financial support, simulation technician availability, and a lack of collaboration with other leading centers to be leading barriers.^9^ This study also demonstrated that difficulty in securing protected time for simulation medical directors, instructor training, and new scenario development were also limitations to simulation implementation, similarly to what was seen in our results.^9^ Median program size did not differ between responding programs with and without SBME.

Our survey revealed integration of simulation into departmental budgets for safety and quality improvement as a relatively unexplored area for funding. Only 11% (7/64) of respondents listed quality improvement as an intended purpose for simulation. Other specialties have leveraged integrating health care simulation as a risk control strategy and have cited a reduction in malpractice premiums from insurers as a way to justify up-front expenses.^13^ Emerging data imply that SBME may reduce errors and improve acute stroke care delivery,^15,16^ potentially improving outcomes and reducing health system costs. Thus, large-scale quality improvement efforts may help convince insurers and health care institutions to financially support simulation efforts in acute neurology. However, such large-scale projects will require collaboration among SBME-using institutions because single centers will likely be underpowered to see such effects.

Such collaborations could also enable impactful and generalizable simulation-based research efforts. This will be paramount in the creation and submission of compelling proposals to granting agencies. Although research grants from the American Board of Psychiatry and Neurology have been previously used to fund neurology resident SBME, collaborative simulation efforts may result in a diversification of funding mechanisms.^17^ The collaborative simulation efforts of the International Network for Simulation-based Pediatric Innovation, Research, and Education (INSPIRE) serve as a potential model, which may lead to a diversification of potential grantors with awards capable of funding neurology simulation initiatives.^18,19^

One potential way to lower the barrier to entry is through shared simulation projects and case exchanges. If made available, it is possible that a collection of templated cases could diminish time simulation faculty spend on case creation. Interest in a neurology simulation case library was expressed by 98% (63/64) of all respondents using simulation at their institution. Collaborative simulation consortiums, such as the INSPIRE group, may inform infrastructure and oversight for a neurology-based simulation case repository.^19^

Ultimately, large-scale adoption of SBME in neurology will be required to develop, standardize, and validate thresholds to demonstrate attainment of ACGME trainee competency milestones.^16,17,20^ Although a lofty goal, having a more objective way to assess competency would allow greater standardization and confidence in trainee's readiness for independent practice.

There are limitations to our study. The survey response rate was suboptimal, with 44% (75/171) contactable neurology residency PDs in the United States. Our results may be biased to programs that do SBME because PDs interested in simulation may have been more likely to complete the survey. It is possible that nonresponding programs may not share similar interests or resources to build simulation programs at their institutions. Surveys were sent from November 2022 to March 2023, which overlapped with residency recruitment timelines and may have affected survey response rates. Our survey did not undergo a formal validation process, although was developed through an iterative process with peer review and consensus among a nation-wide writing committee of SBME-based educators.

A fraction of surveys were incomplete, particularly from programs that do not use or rarely use SBME. As such, the survey may not fully reflect nonrespondent program sizes, interest in simulation growth at their institutions, and access to simulation facilities. Similarly, the survey may not have captured all the potential barriers to using simulation. Owing to incomplete surveys from programs not using SBME, we are limited in our ability to compare barriers with SBME between programs that do and do not use SBME. Although a lack of institutional support was cited as a common barrier limiting simulation use, the exact ways in which an institution may support simulation use were not fully elucidated (access to a center, simulation administrators, funding, etc). Although our survey assessed lack of trained faculty in SBME as a potential barrier to SBME utilization, it did not collect data regarding presence of trained faculty at sites using SBME as a whole. It is possible that SBME is delivered at some institutions without the benefit of trained simulation educators. Structured interviews with select residency PDs regarding their SBME use in residency may provide a more exploratory, granular analysis of these issues. Questions of institutional location, size, ownership, financing, and staffing were not asked in this iteration of our anonymous survey. The upcoming 2023 AAMC medical simulation in medical simulation survey may shed more light on these details.

SBME is an integral part of general medical training, which has been incompletely adopted by neurology residency training programs. The scope of educational topics has been mostly limited to acute care neurology, and there are opportunities to expand the use of simulation beyond clinical education. Neurology residency PDs demonstrated enthusiasm for expanding the use of simulation but are limited by faculty time constraints and deficient institutional support. Collaborative solutions to overcome these barriers are needed to help grow the future directions for SBME within neurology.

## Appendix Authors

**Table:**

Name	Location	Contribution
Shivani Ghoshal, MD	Department of Neurology, Columbia University Vagelos College of Physicians and Surgeons, New York, NY	Drafting/revision of the manuscript for content, including medical writing for content; major role in the acquisition of data; study concept or design; analysis or interpretation of data
Catherine S.W. Albin, MD	Department of Neurology, Emory University School of Medicine, Atlanta, GA	Drafting/revision of the manuscript for content, including medical writing for content; major role in the acquisition of data; study concept or design; analysis or interpretation of data
Nisha A. Malhotra, MD, MPH	Department of Neurology, NYU Grossman School of Medicine, New York	Drafting/revision of the manuscript for content, including medical writing for content; major role in the acquisition of data; analysis or interpretation of data
Ifeyinwa Asonye, MD	Department of Neurology, Columbia University Vagelos College of Physicians and Surgeons, New York, NY	Drafting/revision of the manuscript for content, including medical writing for content; major role in the acquisition of data; study concept or design; analysis or interpretation of data
John Budrow, MD	Department of Neurology, Columbia University Vagelos College of Physicians and Surgeons, New York, NY	Drafting/revision of the manuscript for content, including medical writing for content; analysis or interpretation of data
Rebecca Stainman, MD	Department of Neurology, Johns Hopkins School of Medicine, Baltimore, MD	Drafting/revision of the manuscript for content, including medical writing for content; major role in the acquisition of data; study concept or design; analysis or interpretation of data
Arielle Kurzweil, MD	Department of Neurology, NYU Grossman School of Medicine, New York	Drafting/revision of the manuscript for content, including medical writing for content; major role in the acquisition of data; study concept or design; analysis or interpretation of data
Michelle Bell, MD	Department of Neurology, Columbia University Vagelos College of Physicians and Surgeons, New York, NY	Drafting/revision of the manuscript for content, including medical writing for content; major role in the acquisition of data; study concept or design; analysis or interpretation of data
Jenna Ford, MD	Department of Neurology, Northwestern University Feinberg School of Medicine, Chicago, IL	Drafting/revision of the manuscript for content, including medical writing for content; major role in the acquisition of data
Inna Kleyman, MD	Department of Neurology, Columbia University Vagelos College of Physicians and Surgeons, New York, NY	Drafting/revision of the manuscript for content, including medical writing for content; major role in the acquisition of data
Nina Massad, MD	Department of Neurology, University of Miami Leonard M. Miller School of Medicine, FL	Drafting/revision of the manuscript for content, including medical writing for content; major role in the acquisition of data; analysis or interpretation of data
Yara Mikhaeil-Demo, MD	Department of Neurology, Northwestern University Feinberg School of Medicine, Chicago, IL	Drafting/revision of the manuscript for content, including medical writing for content; major role in the acquisition of data
Briana Wasserstrom, DO	Department of Neurology, University of Florida College of Medicine, Gainesville	Drafting/revision of the manuscript for content, including medical writing for content; major role in the acquisition of data
Zahari Tchopev, MD	Department of Neurology, F. Edward Hebert School of Medicine, Uniformed Services University, Bethesda, MD	Drafting/revision of the manuscript for content, including medical writing for content; major role in the acquisition of data; study concept or design; analysis or interpretation of data
Nicholas A. Morris, MD	Department of Neurology, University of Maryland School of Medicine, Baltimore	Drafting/revision of the manuscript for content, including medical writing for content; major role in the acquisition of data; study concept or design; analysis or interpretation of data

## Glossary

AAMC: Association of American Medical Colleges
ACGME: Accreditation Council for Graduate Medical Education
INSPIRE: International Network for Simulation-based Pediatric Innovation, Research and Education
PD: program director
SBME: simulation-based medical education
